# Detection of CWD Prions in Urine and Saliva of Deer by Transgenic Mouse Bioassay

**DOI:** 10.1371/journal.pone.0004848

**Published:** 2009-03-18

**Authors:** Nicholas J. Haley, Davis M. Seelig, Mark D. Zabel, Glenn C. Telling, Edward A. Hoover

**Affiliations:** 1 Department of Microbiology, Immunology, and Pathology, College of Veterinary Medicine and Biomedical Sciences, Colorado State University, Fort Collins, Colorado, United States of America; 2 Department of Molecular Biology and Genetics, University of Kentucky, Lexington, Kentucky, United States of America; National Institutes of Health, United States of America

## Abstract

Chronic wasting disease (CWD) is a prion disease affecting captive and free-ranging cervids (e.g. deer, elk, and moose). The mechanisms of CWD transmission are poorly understood, though bodily fluids are thought to play an important role. Here we report the presence of infectious prions in the urine and saliva of deer with chronic wasting disease (CWD). Prion infectivity was detected by bioassay of concentrated, dialyzed urine and saliva in transgenic mice expressing the cervid PrP gene (Tg[CerPrP] mice). In addition, PrP^CWD^ was detected in pooled and concentrated urine by protein misfolding cyclic amplification (PMCA). The concentration of abnormal prion protein in bodily fluids was very low, as indicated by: undetectable PrP^CWD^ levels by traditional assays (western blot, ELISA) and prolonged incubation periods and incomplete TSE attack rates in inoculated Tg(CerPrP) mice (373^±^3days in 2 of 9 urine-inoculated mice and 342^±^109 days in 8 of 9 saliva-inoculated mice). These findings help extend our understanding of CWD prion shedding and transmission and portend the detection of infectious prions in body fluids in other prion infections.

## Introduction

Chronic wasting disease (CWD) is an efficiently transmitted prion disease of cervids (e.g. deer, elk, and moose) and is the only prion disease affecting free-ranging, non-domestic animals. The origins of CWD are uncertain, but the disease has been present in wild cervid populations of northern Colorado and southern Wyoming for at least 40 years [1], [2]. Since its discovery, CWD has been identified in captive and free-ranging cervids in 15 states, 2 Canadian provinces, and Korea [3]. As surveillance efforts have intensified, CWD has been detected in areas previously thought to be free of infection, including recent discoveries in West Virginia, New York, and Michigan. The prevalence of CWD varies across North America, but can be as high as 30% in some areas of Colorado [4].

The mechanisms of CWD transmission are not well understood, although there is evidence that infection is transmitted horizontally and can be acquired from environmental sources [5], [6], which underlies the assumption that shedding of infectious prions must be significant. With the expanded recognition of the disease across the continental United States, it is also likely that substantial human exposure has occurred. Nevertheless, because of an apparently strong species barrier [7] and the as yet incompletely understood natural routes and kinetics of CWD transmission, the magnitude and consequence of this exposure remain speculative.

Infectious CWD prions have been detected in saliva and blood, e.g. “prionsialia” and “prionemia,” suggesting a role for specific body fluids in transmission and dissemination [6]. In these bioassay studies in deer, infectivity in urine and feces could not be demonstrated, seemingly at odds with indirect evidence for environmental persistence of CWD prions [5], [8]. In that the presence of prions in body fluids, once thought not to occur, now has impact in understanding of prion transmission and biocontainment, we have continued and extended our investigation of this subject with the present studies.

## Materials and Methods

### Cervid sources

Samples of urine and saliva were collected at terminal disease from five experimentally infected white-tailed deer. Deer in this cohort had been inoculated intracranially with brain, intravenously with blood, or orally with saliva from CWD-infected deer. All source deer were in the terminal stages of CWD infection, and demonstrated moderate to severe neurologic signs, paradoxical polyphagia with declining body condition, polydypsia and polyuria, and were confirmed CWD+ by western blot and immunohistochemistry, as previously described [6]. Histopathologic examination revealed mild nephritis in all the source deer. In some cases, these changes were age related, while in a single case there was appreciable evidence of pyelonephritis. Four of five the deer were homozygous for glycine at amino acid 96 of the cervid prion gene, while one deer was heterozygous at that location, with alleles encoding for both glycine and serine [6].

### Cervid PrP transgenic mice

Tg[CerPrP] line 1536 (*tg1536*) mice were generated in the Telling laboratory at the University of Kentucky [9]. All mice were screened at weaning for the presence of the [CerPrP] construct by conventional and real-time PCR. All mice testing negative for PrP^CWD^ at the completion of bioassay studies were rescreened to reconfirm the presence of cervid PrP gene. Animals were treated according to Colorado State University guidelines.

### Study samples and preparation

A 1% w/v homogenate of brain from a single CWD-positive mule deer (provided by Dr. Michael Miller, Colorado Division of Wildlife) was used as the positive control material. A 1% w/v homogenate of brain from a single CWD-negative white-tail deer from outside the CWD-endemic zone was used as a negative control and was provided by David Osborne, University of Georgia. Urine and saliva samples were collected from five symptomatic whitetail deer as described above.

Spiked positive and negative control samples consisted of 1% homogenates of positive or negative deer brain prepared using 10 ml of negative control saliva or urine as diluent. For the study groups, 10 mls of either urine or saliva were collected from each of five symptomatic deer, 50 ml total volume, and homogenized. All urine and saliva homogenates were then prepared for bioassay by lyophilization followed by resuspension in 0.1 volumes of phosphate-buffered saline (PBS) (e.g. a 10-fold concentration) and dialyzed against 2000 volumes of PBS to return the sample to isotonicity.

### Mouse bioassays

Mice were anesthetized with ketamine and xylazine and inoculated intracerebrally into the left parietal lobe with 30 µl of inoculum. Incubation time was defined as the number of days from inoculation to the onset of clinical neurological signs consistent with a TSE [10]. Animals were euthanized when either a symptomatic TSE or signs of distress were evident. Brains were harvested at necropsy and divided longitudinally, with one half prepared for evaluation by western blotting and PMCA, while the remaining half was fixed in 10% neutral-buffered formalin for histopathology and immunohistochemistry.

### Western blotting (WB)

Brain tissue was initially prepared as a 10% (w/v) suspension in homogenization buffer (150 mM NaCl, 5 mM EDTA, and 1%[v/v] triton-X 100 in PBS). Eleven µl of sample homogenate were mixed with 7 µl of sample buffer (0.1% [v/v] triton-X 100 and 4%(w/v) SDS in PBS) and digested with 2 µl proteinase-K at 500 µg/ml (final concentration: 50 µg/ml) for 20′ at 37°C followed by 10′ at 45°C. Seven µl of 4× running buffer were then added to the sample, followed by denaturation for 5′ at 95°C. Twenty µl of this preparation were run on a pre-cast 12% SDS-PAGE gel (Invitrogen) in a Bio-Rad electrophoresis apparatus for 2 hours at 110 mV. Samples were then transferred to a PVDF membrane for 1 hour at 110 mV in a Bio-Rad transfer apparatus. PVDF membranes were subsequently blocked for 1 hour in 5%(w/v) powdered milk in TBST, followed by application of the primary antibody, BAR224-HRP, diluted 1∶20,000 in 5% powdered milk in TBST, for 1 hour. Following washing, immunoreactivity was detected using an enhanced chemiluminescent detection system (ECL-plus, Amersham Biosciences) in an LAS 3000™ imaging system. (Fuji Photo Film, Fuji Inc, Valhalla, NY)

### Histopathology and Immunohistochemistry (IHC)

Cervid renal tissues and *tg1536* neural tissues were fixed in formalin overnight, treated with 88% formic acid for one hour, washed in tap water for two hours, and then stored in 60% ethanol prior to paraffinization. Paraffin-embedded tissue sections (6 µm) were mounted onto positively charged glass slides, deparaffinized, and rehydrated through graded ethanol. Tissues were subjected to Heat Induced Epitope Retrieval (HIER) using an automated antigen-retrieval system (Retriever™) and a proprietary buffer solution (DakoCytomation Target Retrieval Solution, DAKO, Hamburg, Germany). Tissues were then stained with an automated immunostainer, using PrP monoclonal antibody BAR-224 conjugated to HRP as the primary antibody (1∶250 final dilution). Detection was completed using HRP-mediated hydrogen peroxide immunostaining (AEC+, DAKO), with haematoxylin as a counterstain.

### Protein Misfolding Cyclic Amplification (PMCA)

Source inocula as well as mice negative for PrP^CWD^ by both WB and IHC were further analyzed by PMCA. The amplification protocol, described below, was similar to those described by Soto and colleagues [11], [12]. Normal brain homogenate (NBH), the substrate for prion conversion *in vitro*, was prepared in a room that had not previously been used for prion research as follows: naïve *tg1536* mice were euthanized intraperitoneally with 15 mg of sodium pentobarbital and perfused with 25 ml of 5 mM EDTA in PBS via intracardiac catheterization. The calvarium was removed and the entire brain excised and placed on ice. Brain homogenate was then prepared at a 10% (w/v) solution in PMCA buffer (1% triton-X 100 [v/v], 5 mM EDTA, 150 mM NaCl, and 0.5% saponin [w/v] in PBS adjusted to a pH of 7.2) with the addition of Complete Protease Inhibitors (Roche Pharmaceuticals, Indianapolis, IN) in a dounce homogenizer. Homogenates were then centrifuged for 1 minute at 2000 rpm to remove bulk brain material, and the supernatant frozen in single-experiment aliquots at −70°C in a “prion-free” room until use in PMCA. Experimental handling protocols were identical to protocols commonly used for PCR: NBH was added to a plate in a room not previously used for prion research and then transferred to a room used exclusively for prion research, where samples were added in a biosafety hood prior to sonication. Twenty-five µl of either source inocula or WB and IHC negative brain homogenate was added to 25 µl of NBH in individual wells of a 96 well PCR plate (USA Scientific, Ocala, FL), placed in an ultrasonic processor (Misonix, Farmingdale, NY) and incubated at 37°C. Samples were sonicated for 40 s at power setting 7.0, followed by 30 minutes of incubation. Ninety six cycles of sonication were preformed over 48 hours, with a 25 µl aliquot transferred to a fresh NBH preparation for serial amplification. Following three rounds of amplification, samples were evaluated by western blotting, as described above, for the presence of PrP^CWD^.

## Results

To investigate whether urine may play a role in natural CWD transmission, and to confirm the presence of PrP^CWD^ in saliva, we pooled and concentrated urine or saliva from five terminally infected CWD+ deer and inoculated two groups of *tg1536* mice. Following inoculation, mice were monitored for clinical signs of prion infection and were euthanized when terminal disease was apparent. Central nervous system tissues were evaluated for PrP^CWD^ using western blotting (WB), immunohistochemistry (IHC) and, when negative by conventional assays, protein misfolding cyclic amplification (PMCA).

In a group of 9 mice inoculated with lyophilized urine, 2 animals developed neurologic disease consistent with a TSE (Table 1), including ataxia, a slow, lumbering gait, and poor thrift, at 370 and 376 days post-inoculation (dpi). Eight out of 9 mice inoculated with prepared saliva likewise developed signs of TSE at 342+/−109 dpi. All mice in positive control groups, inoculated with CWD+ brain spiked into either urine or saliva, developed disease before 370 dpi (235+/−91 dpi), while none of the mice in either negative control group, inoculated with either urine or saliva spiked with negative brain homogenate, demonstrated clinical evidence of a TSE after >640 days.

**Table 1:** Western Blot (WB), immunohistochemistry (IHC), and protein-misfolding cyclic amplification results and incubation periods of Tg[CerPrP] mouse bioassay.

Mouse Bioassay				
Inoculum	WB+	IHC+	PMCA+	Incubation Period
**(+) Control**	18/18	18/18	N.A.	235+/−91d
**Urine**	2/9	2/9	1/7	373+/−3d
**Saliva**	8/9	8/9	0/1	342+/−109d
**(−) Control**	0/18	0/18	0/18	>640d

Numerators indicate the number of animals testing positive by a particular assay, while denominators designate the total number tested. PMCA analysis was reserved for mice testing negative by traditional assays. Incubation periods indicate the survival times in days post inoculation +/− one standard deviation. N.A. - not assayed.

### WB and IHC results

Brains of all mice demonstrating terminal neurologic disease, including 2 of 9 inoculated with urine and 8 of 9 inoculated with saliva, had evidence of protease-resistant prion protein by both WB and IHC. PrP^CWD^ was absent by both WB and IHC in mice not displaying clinical disease, including those in negative control groups.

**Figure 1:**
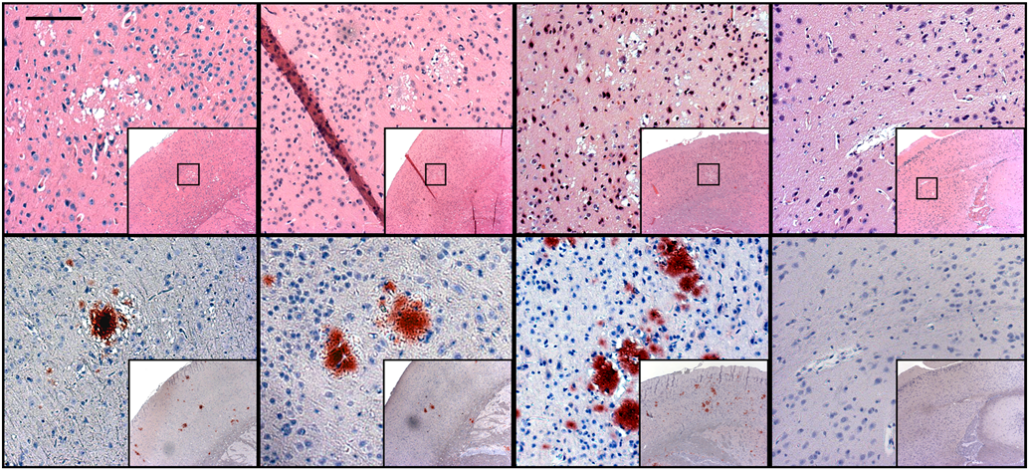
Spongiform degeneration and PrP^CWD^ identified by histopathology and immunohistochemistry. Vacuolated neurons and spongiform degeneration of the neuropil characteristic of a TSE is evident on H&E staining, with the colocalization of PrP^CWD^ specific immunostaining of florid plaques in the cortices of mice inoculated with positive control inoculum and concentrated urine and saliva from CWD-infected cervids. Negative control mice showed no evidence of spongiform degeneration or PrP^CWD^ immunostaining. HRP-conjugated BAR-224 was used as a primary antibody. (Measure bar, 50 µm).

Immunohistochemistry demonstrated widely distributed, florid PrP^CWD^ plaques, with no apparent relationship between deposition pattern, lesion severity, and source inoculum (Figure 1). In cases with the least severe pathology, however, cortical lesions predominated; with increasing neuropathology, lesions were further distributed within the hippocampus, midbrain, and cerebellum.

**Figure 2:**
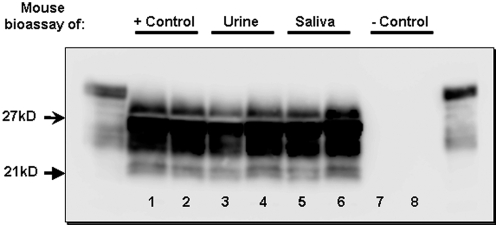
Western Blot detection of PrP^CWD^ in urine and saliva-inoculated mice. Western blotting analysis of control and test mice, demonstrating PrP^CWD^ in positive control mice (lanes 1 and 2), as well as urine (lanes 3 and 4) and saliva (lanes 5 and 6) inoculated mice. Protease-resistant prions were not detected in negative control mice (lanes 7 and 8). Flanking lanes represent undigested PrP^C^.

In western blotting, PrP^CWD^ proteinase K-resistant glycoforms spanned 21–27 kD. In all cases, the dominant PrP^CWD^ glycoform was the di-glycosylated band, followed by mono- and non-glycosylated isoforms (Figure 2).

**Figure 3:**
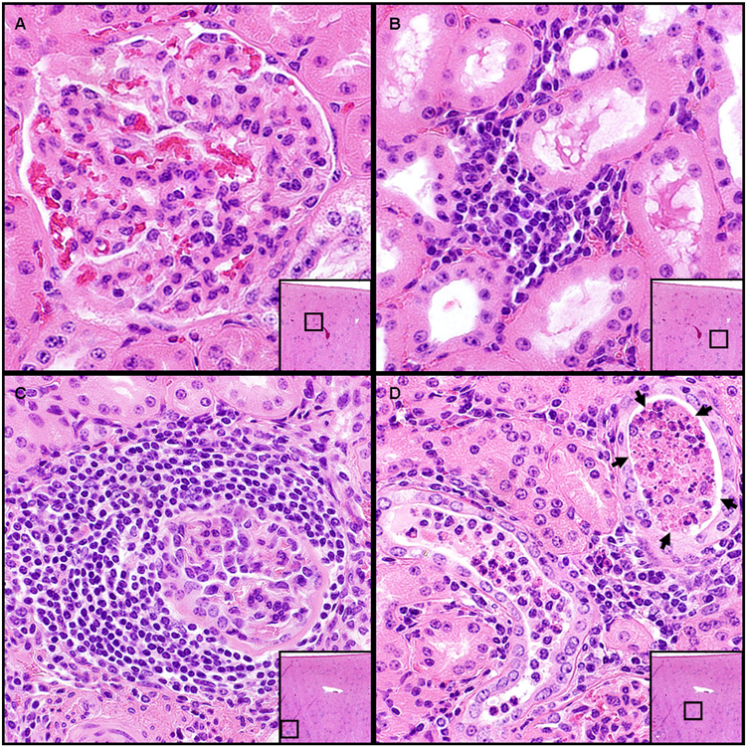
Histopathologic evaluation of renal tissues from donor cervids. (A) Minimal, chronic and proliferative glomerular disease and (B) mild interstitial fibrosis and lymphocytic infiltration were observed in 4 out of 5 donor deer. The remaining deer showed evidence of mild lymphocytic glomerulonephritis (C) as well as “tubular proteinosis.” (D, arrows).

To identify potential pathological mechanisms for prionuria, histopathologic examination of donor renal tissues was also performed. Microscopic evaluation of H&E stained kidney sections from each of the donor deer revealed minimal histologic disease in 4 of the 5 animals. Lesions in these animals were characterized by the combination of minimal proliferative glomerular disease and mild interstitial fibrosis and lymphocytic inflammation (Figure 3A and B). In these animals, there was no appreciable histologic evidence of proteinuria or pyelonephritis. In the fifth animal, more significant renal pathology was detected. In this animal, there was a combination of mild, chronic, lymphocytic glomerulonephritis, which was similar to the previous 4 animals, and a moderately severe, chronic, lymphocytic interstitial nephritis with light microscopic evidence of renal protein loss (“tubular proteinosis”). (Figures 3C and D).

### PMCA

**Figure 4:**
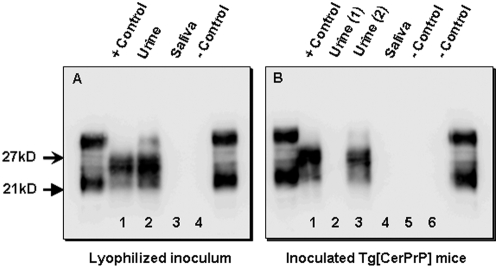
Serial PMCA amplification of PrP^CWD^ in concentrated deer urine and in the brains of urine-inoculated mice. A) PrP^CWD^ was detectable by serial PMCA (sPMCA) in control and urine inocula (lanes 1 and 2, respectively), while PrP^CWD^ could not be identified in saliva and negative control inocula (lanes 3 and 4, respectively) after 3 rounds of amplification. B) Three rounds of sPMCA also amplified PrP^CWD^ in the brains of CWD-infected mice, including positive-control inoculated mice and a single mouse inoculated with lyophilized urine (lanes 1 and 3, respectively). PrP^CWD^ was not amplified in mice inoculated with negative control material (lanes 5 and 6) or in other mice inoculated with either urine (lane 2) or saliva (lane 4) from CWD+ deer. All flanking lanes represent undigested PrP^C^.

Concentrated samples used for mouse inoculation were assayed for PrP^CWD^ by serial PMCA (sPMCA) over three rounds of amplification. In our experience, three rounds of amplification permits an approximate 4000-fold increase in sensitivity as compared to traditional western blotting detection, while avoiding both cross-contamination and generation of any spontaneously formed protease-resistant PrP, thereby maintaining 100% specificity [13]. In three independent experiments, PrP^CWD^ was identified in lyophilized urine homogenate from CWD+ deer. (Figure 4A) PrP^CWD^ was also found in both positive control inocula after the initial round of amplification, while PrP^CWD^ was not detected in either saliva or negative control preparations.

To increase detection sensitivity in bioassay experiments, brains from all mice that tested negative for PrP^CWD^ by WB and IHC, including negative controls, were re-evaluated by sPMCA. The brain from one WB− and IHC-negative mouse that had been inoculated with urine from a CWD+ deer and expired at 582 dpi amplified PrP^CWD^ in three independent PMCA experiments (Figure 4B). No mice in either of the negative control groups were positive using this assay.

## Discussion

The salient feature of chronic wasting disease is its facile transmission among its host species. Until recently, little was known regarding the mechanisms of this efficient transmissibility, however, we have previously demonstrated infectious prions in the saliva and blood of infected deer [6]. By using intracerebral inoculation of concentrated urine in cervid PrP transgenic mice, we report the presence of infectious prions in urine from CWD-infected cervids, and confirm the phenomenon of prionsialia in these animals. The identification of CWD prions in bodily fluids described in the current report could portend infectivity in secretions and excretions in other prion diseases.

In contrast to the data presented here, oral inoculation of urine in cervid bioassays was unable to identify infectious prions in the urine of CWD+ deer [6]. This result could have been due to necessarily limited observation period possible in those studies (18 months), or variations in source and recipient genotype [14], [15], route of inoculation [16], or the sensitivity of traditional PrP^CWD^ detection assays [17], [18]. The mule deer providing inoculum pools in prior studies were of an unreported genotype; the majority of the recipient deer were homozygous for glycine at residue 96, although a single animal was heterozygous; sharing both G96 and S96 alleles [6]. Likewise, the inocula used in the present study were pooled from sources heterogeneous at codon 96 of the cervid prion gene. Transgenic mice used in bioassay studies, on the other hand, were uniformly homogenous for a glycine residue at this position [9], a polymorphism which is reported to be overrepresented in CWD-infected deer [19]. As a result, it is possible that the genotypic background of either source or subject animals may have been a factor in susceptibility, though we are at present unable to draw any concrete conclusions regarding this relationship. While mouse genotype may have played a role in the outcome, it is also probable that cervid PrP transgenic mouse bioassay simply represents a more sensitive detection system for prions in excreta. Intracranial inoculation, reportedly a more sensitive route of prion exposure [16], [20], is more easily performed in mouse bioassay, a model which also permits extended incubation periods and inclusion of a greater number of test animals.

While our findings point to urine as an additional vehicle for CWD transmission, only 2 of 9 inoculated *tg1536* mice were confirmed WB/IHC-positive for prion infection, with a third PrP^CWD^+ animal later identified by PMCA. This contrasts with 8 of 9 positive mice receiving saliva and infers a much lower concentration of prion infectivity in urine. The wide range of survival times in inoculated mice suggests relatively low levels of infectious prions and/or uneven distribution of infectious PrP moieties in the inocula [21]. Differing [CerPrP] zygosity in *tg1536* mice (homozygous vs. hemizygous) may also have played a role in this variation.

Using sPMCA, PrP^CWD^ was repeatedly identified in test urine and spiked urine and saliva used as positive control, but was not detected in test saliva after three rounds of amplification. The reasons for our inability to identify PrP^CWD^ in saliva – given the definitive bioassay findings – remain unknown, and we propose the presence of as-yet unidentified inhibitors such as mucin or salivary proteases which are thought to negatively affect other *in vitro* assays [22], [23].

The finding of PrP^CWD^ in urine and saliva calls for the identification of the pathological processes and cellular associations of the prion protein involved in shedding. Previous studies have related renal pathology to prionuria [24], [25], a finding which corresponds to our identification of mild to moderate nephritis in those deer providing samples for the current study. It is plausible that renal pathology contributed to prionuria in each of these animals; as samples were pooled, however, we cannot identify specific animals in which it may have been occurring, nor can we accurately estimate the relative level of prionuria occurring in each donor as ultrastructural studies were not performed [26]. While we have not yet identified pathologic prions in renal source tissues [Unpublished data], protease-resistant PrP^CWD^ has been identified by immunostaining in renal tissue of prion-infected deer [27], sheep [28], hamsters and most intriguingly humans [29], foreshadowing the potential for prionuria in other transmissible spongiform encephalopathies. We continue to examine tissues from CWD+ deer in an effort to determine the pathogenesis and kinetics of CWD prion excretion and shedding.

Evidence for excretion and shedding of infectious prions is also accumulating in the scrapie system. PrP^C^-converting activity has been identified by sPMCA in the urine of scrapie-infected sheep, hamsters and mice [21], [30], [31], [32]. Prion infectivity has also been demonstrated in the feces of hamsters orally infected with scrapie [33]. Other studies point to infectious prions in the milk of scrapie-infected ewes [34], [35]. As noted above, it remains unknown whether other prion diseases (e.g. Kuru, BSE, CJD, TME) may be transmitted by bodily fluids or excreta other than blood. Additional studies examining feces, milk, and other body fluids are therefore necessary in CWD and other prion diseases, studies currently underway in our laboratory.

As CWD transmission may model communicability of other TSE's, the transmissible nature of prion diseases *may* serve as a model for other protein-misfolding diseases. For example, feces, but not urine, from both mice and cheetahs affected with systemic amyloidosis A (SAA) was recently shown to induce SAA in a mouse model, although negative controls were not available in those studies [36]. In light of the prionuria detected in CWD and in models of scrapie, further investigations of infectivity in body fluids in other protein folding diseases may be warranted in the event that prion diseases are not the only infectious proteinopathies.

In summary, we confirm prionsialia in CWD-affected deer by bioassay in cervidized mice and demonstrate for the first time infectious prions in the urine of these cervids by both bioassay and sPMCA. We are currently evaluating urine and saliva from individual animals in hopes of identifying predisposing factors, such as genotypic background and underlying pathology, which may contribute to prionuria and prionsialia. Concurrently, we have begun to explore the tissue origins and protease sensitivity of the infectious prions as well as the onset and duration of shedding in these bodily fluids.

## References

[1] Williams ES, Young S (1980). Chronic wasting disease of captive mule deer: a spongiform encephalopathy.. J Wildl Dis.

[2] Williams ES, Young S (1982). Spongiform encephalopathy of Rocky Mountain elk.. J Wildl Dis.

[3] Sigurdson CJ (2008). A prion disease of cervids: chronic wasting disease.. Vet Res.

[4] Williams ES (2005). Chronic wasting disease.. Vet Pathol.

[5] Miller MW, Williams ES, Hobbs NT, Wolfe LL (2004). Environmental sources of prion transmission in mule deer.. Emerg Infect Dis.

[6] Mathiason CK, Powers JG, Dahmes SJ, Osborn DA, Miller KV (2006). Infectious prions in the saliva and blood of deer with chronic wasting disease.. Science.

[7] Kong Q, Huang S, Zou W, Vanegas D, Wang M (2005). Chronic wasting disease of elk: transmissibility to humans examined by transgenic mouse models.. J Neurosci.

[8] Williams ES, Young S (1992). Spongiform encephalopathies in Cervidae.. Rev Sci Tech.

[9] Browning SR, Mason GL, Seward T, Green M, Eliason GA (2004). Transmission of prions from mule deer and elk with chronic wasting disease to transgenic mice expressing cervid PrP.. J Virol.

[10] Carlson GA, Kingsbury DT, Goodman PA, Coleman S, Marshall ST (1986). Linkage of prion protein and scrapie incubation time genes.. Cell.

[11] Saborio GP, Permanne B, Soto C (2001). Sensitive detection of pathological prion protein by cyclic amplification of protein misfolding.. Nature.

[12] Soto C, Saborio GP, Anderes L (2002). Cyclic amplification of protein misfolding: application to prion-related disorders and beyond.. Trends Neurosci.

[13] Kurt TD, Perrott MR, Wilusz CJ, Wilusz J, Supattapone S (2007). Efficient in vitro amplification of chronic wasting disease PrPRES.. J Virol.

[14] Hamir AN, Gidlewski T, Spraker TR, Miller JM, Creekmore L (2006). Preliminary observations of genetic susceptibility of elk (Cervus elaphus nelsoni) to chronic wasting disease by experimental oral inoculation.. J Vet Diagn Invest.

[15] O'Rourke KI, Spraker TR, Zhuang D, Greenlee JJ, Gidlewski TE (2007). Elk with a long incubation prion disease phenotype have a unique PrPd profile.. Neuroreport.

[16] Hamir AN, Kunkle RA, Richt JA, Miller JM, Cutlip RC (2005). Experimental transmission of sheep scrapie by intracerebral and oral routes to genetically susceptible Suffolk sheep in the United States.. J Vet Diagn Invest.

[17] Safar JG, Geschwind MD, Deering C, Didorenko S, Sattavat M (2005). Diagnosis of human prion disease.. Proc Natl Acad Sci U S A.

[18] Grassi J, Maillet S, Simon S, Morel N (2008). Progress and limits of TSE diagnostic tools.. Vet Res.

[19] Johnson C, Johnson J, Clayton M, McKenzie D, Aiken J (2003). Prion protein gene heterogeneity in free-ranging white-tailed deer within the chronic wasting disease affected region of Wisconsin.. J Wildl Dis.

[20] Hamir AN, Kunkle RA, Bulgin MS, Rohwer RG, Gregori L (2008). Experimental transmission of scrapie agent to susceptible sheep by intralingual or intracerebral inoculation.. Can J Vet Res.

[21] Kariv-Inbal Z, Ben-Hur T, Grigoriadis NC, Engelstein R, Gabizon R (2006). Urine from scrapie-infected hamsters comprises low levels of prion infectivity.. Neurodegener Dis.

[22] Ochert AS, Boulter AW, Birnbaum W, Johnson NW, Teo CG (1994). Inhibitory effect of salivary fluids on PCR: potency and removal.. PCR Methods Appl.

[23] Archibald DW, Cole GA (1990). In vitro inhibition of HIV-1 infectivity by human salivas.. AIDS Res Hum Retroviruses.

[24] Seeger H, Heikenwalder M, Zeller N, Kranich J, Schwarz P (2005). Coincident scrapie infection and nephritis lead to urinary prion excretion.. Science.

[25] Siso S, Gonzalez L, Jeffrey M, Martin S, Chianini F (2006). Prion protein in kidneys of scrapie-infected sheep.. Vet Rec.

[26] Nacar A, Karabay G, Unlukal N, Yazici C, Ozdemir H (2008). Correlation between proteinuria level and renal morphology with special reference to electron microscopy in kidney donors.. Ren Fail.

[27] Hamir AN, Kunkle RA, Miller JM, Hall SM (2006). Abnormal prion protein in ectopic lymphoid tissue in a kidney of an asymptomatic white-tailed deer experimentally inoculated with the agent of chronic wasting disease.. Vet Pathol.

[28] Ligios C, Cancedda GM, Margalith I, Santucciu C, Madau L (2007). Intraepithelial and interstitial deposition of pathological prion protein in kidneys of scrapie-affected sheep.. PLoS ONE.

[29] Fournier JG, Escaig-Haye F, Billette de Villemeur T, Robain O, Lasmezas CI (1998). Distribution and submicroscopic immunogold localization of cellular prion protein (PrPc) in extracerebral tissues.. Cell Tissue Res.

[30] Murayama Y, Yoshioka M, Okada H, Takata M, Yokoyama T (2007). Urinary excretion and blood level of prions in scrapie-infected hamsters.. J Gen Virol.

[31] Andrievskaia O, Algire J, Balachandran A, Nielsen K (2008). Prion protein in sheep urine.. J Vet Diagn Invest.

[32] Gonzalez-Romero D, Barria MA, Leon P, Morales R, Soto C (2008). Detection of infectious prions in urine.. FEBS Lett.

[33] Safar JG, Lessard P, Tamguney G, Freyman Y, Deering C (2008). Transmission and detection of prions in feces.. J Infect Dis.

[34] Gresham A (2008). Scrapie transmission via milk.. Vet Rec.

[35] Konold T, Moore SJ, Bellworthy SJ, Simmons HA (2008). Evidence of scrapie transmission via milk.. BMC Vet Res.

[36] Zhang B, Une Y, Fu X, Yan J, Ge F (2008). Fecal transmission of AA amyloidosis in the cheetah contributes to high incidence of disease.. Proc Natl Acad Sci U S A.

